# Inflammation as a master regulator of immunotherapy response in head and neck squamous cell carcinoma: from malignant transformation to ecology-aware precision combinations

**DOI:** 10.3389/fimmu.2026.1798938

**Published:** 2026-07-17

**Authors:** Yingchen Han, Peihong Wu, Haoran Song, Ruoxi Yu, Ruotong Liu, Zhenguang Du, Zhaozhe Liu, Jia Gu

**Affiliations:** 1 Department of Medical Oncology, The First Affiliated Hospital of China Medical University, Shenyang, Liaoning, China; 2 Department of Otolaryngology, The First Affiliated Hospital of China Medical University, Shenyang, Liaoning, China; 3 Shenyang Medical College, Shenyang, Liaoning, China; 4 Department of Interventional Medicine, Liaoning Provincial People’s Hospital, Shenyang, Liaoning, China; 5 Department of Oncology, General Hospital of the Northern Theater Command, Shenyang, Liaoning, China

**Keywords:** cancer-associated fibroblasts, head and neck squamous cell carcinoma, immune checkpoint blockade, inflammation, myeloid cells, tumor microenvironment

## Abstract

In the era of immunotherapy, head and neck squamous cell carcinoma (HNSCC) has demonstrated clear benefits from immune-based treatments and is widely regarded as a tumor type with high immunotherapeutic potential. These tumors are characterized by robust and persistent inflammatory responses that actively drive tumor initiation and progression while concurrently shaping their sensitivity or resistance to therapy. Inflammation simultaneously creates therapeutic vulnerabilities and barriers by altering tumor behavior and reprogramming the immune microenvironment. This review examined HNSCC through a tripartite prism of inflammation, immunity, and tumor biology to demonstrate how chronic inflammatory cues rewire immune cells, reshape signaling circuits, and remodel tissue architecture, ultimately altering responses to immunotherapy. We first examined how immune cell reprogramming occurs under inflammatory pressure. Macrophages, regulatory T cells, exhausted CD8^+^ T cells, and specialized dendritic cell subsets can switch roles—from tumor-clearing sentinels to promoters of tumor growth, stemness, and invasion. This plasticity—sometimes transient, sometimes entrenched—determines whether the immune ecosystem favors elimination or tolerance, and consequently whether immune checkpoint blockade succeeds or fails. Next, we catalogued the inflammation-linked pathways and readouts that capture these state changes. Signaling hubs such as NF-κB/STAT3, IL-6/TNFα, TGF-β, HA-CD44, and PI3K-4EBP1-SOX2 orchestrate the trade-offs between proliferation and invasion and govern cancer stem cell dynamics. Corresponding biomarkers—PD-L1, CD163/CD68 ratios, LAMP3, ALDH/SOX2, Zeb1, Vimentin, and CD44 isoforms—become far more informative when resolved at single-cell and spatial scales, thereby enabling sharper patient stratification. We then mapped the pathological interplay among tumor, stromal, and immune compartments. Extracellular matrix reprogramming, CAF heterogeneity, and the spatial polarity of immune infiltrates generate discrete micro-niches with distinct functional consequences. Spatial profiling can convert static pathology into a dynamic atlas of therapeutic opportunities. Finally, we outlined translational directions. Targeting inflammation and the microenvironment—via TAM reprogramming, cytokine blockade, or STING/CD47 pathway modulation combined with immune checkpoint blockade (ICB)—offers a rational strategy to improve outcomes. Altogether, these strategies point toward an ecology-aware approach to precision immunotherapy for HNSCC—one that reads and reshapes the tumor’s inflammatory language rather than ignoring it.

## 1 Introduction

Head and neck cancer (HNC) comprises a heterogeneous group of malignancies arising from the oral cavity, pharynx, larynx, sinonasal tract, and related anatomical sites, among which head and neck squamous cell carcinoma (HNSCC) represents the predominant histological subtype. Recent GLOBOCAN 2022 estimates reported approximately 940,000 new cases and 480,000 deaths from head and neck cancer worldwide, while cancers of the lip, oral cavity, and pharynx accounted for approximately 758,000 new cases; therefore, these population-level estimates should be interpreted according to anatomical and histological scope. Despite advances in multimodal therapy, its prognosis remains poor– overall five-year survival is only on the order of 50% (1, 2). Most patients present with locally advanced disease, and even with aggressive treatment more than half will experience locoregional recurrence or distant metastasis, with a median survival under one year once the cancer progresses (1, 2). HNSCC arises in diverse anatomic subsites (oral cavity, oropharynx, larynx, hypopharynx, etc.) and encompasses etiologically heterogeneous tumors driven by both carcinogen exposure and viral infection (1, 3). Traditional risk factors such as tobacco use and alcohol abuse induce chronic mucosal inflammation that promotes malignant transformation, whereas high-risk human papillomavirus (HPV) infection (primarily sub-type 16) has emerged as a major cause of oropharyngeal carcinoma (4). In recent decades, the incidence of HPV-positive oropharyngeal squamous cell carcinoma has risen substantially, especially among younger patients in several high-income regions; however, HPV attribution should not be generalized to all HNSCC, because HPV-related disease is concentrated primarily in the oropharyngeal subsite and varies substantially by geography and anatomical location. These epidemiological patterns underscore the multi-factorial nature of HNSCC and partly explain the marked molecular and clinical heterogeneity observed between tumors of different sub-sites and etiologies (1). Notably, overall outcomes for advanced HNSCC have improved little over the past 20–30 years, highlighting the urgent need for more effective systemic therapies (1).

Immunotherapy has recently reshaped the therapeutic landscape of HNSCC, following its success in other refractory cancers (5). For many years, the only approved targeted drug in HNSCC was the epidermal growth factor receptor inhibitor cetuximab, underscoring the difficulty of treating this disease with conventional modalities (1). In the past five years especially, immune-based treatments have represented a major breakthrough in oncology, yielding significant clinical benefits across multiple difficult-to-treat malignancies, including HNSCC (5). Monoclonal antibodies blocking the PD-1/PD-L1 immune checkpoint can reinvigorate anti-tumor T cell activity and have demonstrated improved survival in a subset of patients with recurrent or metastatic HNSCC (6). Clinically, nivolumab and pembrolizumab have established approved roles in HNSCC, but their indications are setting-specific. Nivolumab is approved for recurrent or metastatic squamous cell carcinoma of the head and neck after progression on platinum-based therapy (7). Pembrolizumab is approved for first-line metastatic or unresectable recurrent HNSCC and, more recently, for perioperative treatment of resectable locally advanced HNSCC with PD-L1 CPS ≥1 (8). By contrast, atezolizumab remains investigational in HNSCC and should not be described as having an approved HNSCC indication (9). These immunotherapies can induce durable remissions in certain cases, confirming that HNSCC is a tumor type with genuine immunotherapeutic potential (6). Broadly speaking, current immunotherapeutic strategies in HNSCC encompass not only ICB but also tumor vaccines, cytokine therapies, adoptive T cell transfers, and other immune modulators aimed at restoring effective anti-tumor immunity (6). However, the majority of HNSCC patients do not attain lasting benefit from present immunotherapies (5). Both primary resistance (failure to ever respond) and acquired resistance (relapse after an initial response) are frequently observed, limiting long-term success to a minority of cases (5). In addition, multiple studies have reported an atypical phenomenon of “hyperprogressive disease” in which tumors paradoxically accelerate their growth upon PD-1/PD-L1 blockade (10). This alarming hyper-progression, although relatively rare, is associated with significantly worse outcomes and is suspected to represent an immune-related adverse effect of checkpoint inhibition (10). These clinical realities underscore that while unleashing an anti-tumor immune response can produce dramatic regressions, intrinsic escape mechanisms and dynamic tumor–immune interactions ultimately determine immunotherapy efficacy (11).

Chronic inflammation is now recognized as a key driver of HNSCC carcinogenesis and a central modifier of treatment responses. Persistent carcinogen- and virus-related inflammatory stimuli—including tobacco smoke exposure, alcohol-associated epithelial injury and oxidative stress, and high-risk HPV infection—can activate NF-κB/STAT3-centered inflammatory programs, cytokine release, and immune reprogramming, thereby creating a pro-tumorigenic microenvironment that fosters the stepwise transformation of normal epithelium into cancer (3, 4, 9). HNSCC often develops through pre-malignant lesions under the impetus of sustained inflammation and oxidative stress (12, 13). Indeed, tumor-promoting inflammation has been established as an “enabling” hallmark of cancer that fuels multiple malignant traits, from genomic instability to angiogenesis and metastasis (14). In the head and neck, smoking- and alcohol-associated cancers typically arise against a backdrop of chronic mucosal inflammation, whereas HPV-driven tumors originate from virus-induced dysplasia– distinct etiologic pathways that both exemplify inflammation-driven tumorigenesis (4). Notably, although histopathological progression from dysplasia to carcinoma *in situ* to invasive carcinoma can often be observed, many patients present with advanced HNSCC without any known precancerous phase (4, 15). This suggests that inflammation-mediated genetic and epigenetic reprogramming may accelerate “field cancerization,” allowing malignancy to emerge covertly and complicating early detection (4). Moreover, HNSCC tumors are generally infiltrated by a rich array of immune cells as a consequence of these chronic inflammatory conditions, and the composition of this immune infiltrate is a pivotal determinant of tumor behavior and therapy response (1, 16).

Within the inflamed tumor microenvironment (TME) of HNSCC, the immune system’s role becomes paradoxical. On one hand, immune surveillance can recognize and eliminate emerging tumor cells, suppressing malignancy; on the other hand, chronic inflammation can re-educate immune cells toward pro-tumor phenotypes, enabling cancer progression and immune evasion (1). It is now understood that immunity exerts a dual influence on tumor development: it not only protects the host by destroying nascent cancer cells, but also selects for more resistant clones and helps construct a tumor-permissive niche in which those cells can thrive (11). This concept is captured by the cancer immunoediting model, in which tumor–immune interactions proceed through elimination, equilibrium, and escape phases, explaining how immune pressure can both suppress tumor emergence and sculpt immune-resistant malignant clones (17). In HNSCC, continuous inflammatory signaling skews the phenotype of infiltrating leukocytes, shifting the local balance from anti-tumor immunity toward pro-tumor immune tolerance (1, 18). Tumor-associated macrophages (TAMs), regulatory T cells, exhausted T cells and other immunosuppressive populations accumulate in chronically inflamed head and neck tumors (1). Through the release of anti-inflammatory cytokines like IL-10 and TGF-β and the up-regulation of inhibitory ligands such as PD-L1, these cells create a tolerogenic milieu that blunts natural immune surveillance. As a result, HNSCC often exhibits an “immune-cold” or immune-excluded phenotype in which effector T cells are either rendered dysfunctional or physically sequestered, a state correlated with poor responsiveness to PD-1/PD-L1 blockade therapy (1, 19). This association is consistent with immune-subgroup analyses showing that HNSCC tumors differ markedly in inflammatory activity, cytolytic signaling, stromal exclusion, and exhausted immune states. In this context, insufficient effector T-cell infiltration, low T-cell-inflamed/IFN-γ signaling, or spatial exclusion of lymphocytes may explain why some tumors remain refractory to ICB despite the presence of checkpoint targets (20, 21). In sum, chronic inflammation not only initiates and promotes HNSCC at the genetic and molecular level, but also orchestrates an immunosuppressive microenvironment that allows tumor cells to escape host immunity and resist immunotherapeutic attack (18, 22).

Confronted with the intertwined challenges of inflammation-driven tumor progression and immune evasion, the current era of HNSCC research is increasingly focused on integrated therapeutic strategies. It has become evident that no single modality is sufficient for most patients– combination approaches are required to overcome resistance mechanisms and achieve durable control (23). Immune checkpoint inhibitors are now being combined with other treatments– such as adoptive T cell therapy, cancer vaccines, cytokine blockers, and conventional modalities (surgery, radiation, chemotherapy)– to exploit synergistic effects (23). Targeting the inflammatory TME itself is a promising avenue: for example, reprogramming TAMs from an M2 (tumor-promoting) state toward an M1 (tumoricidal) phenotype, or neutralizing chronic inflammatory mediators like IL-6 and TGF-β, may help break the cycle of immune suppression and restore anti-tumor reactivity (24). The incorporation of predictive biomarkers to stratify patients– distinguishing likely responders from non-responders– and close monitoring of immune-related adverse events are also critical to maximizing safety and efficacy (23). Ultimately, a deeper understanding of how chronic inflammation, immune dysfunction, and tumor biology intersect in HNSCC will pave the way for more effective personalized therapies (18, 25).

In this review, we therefore adopt a tripartite perspective encompassing inflammation, immunity, and tumor biology to explore HNSCC (26, 27). We examine how persistent inflammatory signals can rewire immune cell function, remodel signaling pathways and tissue architecture, and ultimately influence immunotherapy outcomes (19, 28). By elucidating the mechanisms of inflammation-driven malignant transformation and immune escape in HNSCC, our goal is to highlight new opportunities to improve immunotherapeutic responses and patient prognosis (11, 29).

## 2 Literature search strategy

This article was designed as a narrative review rather than a systematic review or meta-analysis. To improve transparency and reduce the risk of selective citation, we performed a structured literature search of PubMed/MEDLINE, Web of Science, Scopus, and Embase from database inception to June 3, 2026. Additional sources included ClinicalTrials.gov, FDA approval information, major oncology guidelines, and reference lists of highly relevant reviews and primary studies.

The search strategy combined terms related to disease context, inflammatory biology, immune microenvironment, biomarkers, and treatment response, including “head and neck squamous cell carcinoma,” “HNSCC,” “oral squamous cell carcinoma,” “OSCC,” “oropharyngeal squamous cell carcinoma,” “HPV,” “p16,” “inflammation,” “chronic inflammation,” “TME,” “ICB,” “PD-1,” “PD-L1,” “PD-L1 CPS,” “NF-κB,” “STAT3,” “TGF-β,” “macrophage,” “monocyte,” “myeloid-derived suppressor cell,” “neutrophil,” “VISTA,” “TIM-3,” “STING,” “CXCL9,” “CXCL10,” “CXCL11,” “CXCR3,” “CD103 dendritic cell,” “spatial transcriptomics,” “single-cell RNA sequencing,” “tumor-infiltrating lymphocytes (TILs),” “TAM/CD8^+^ T-cell ratio,” “tumor mutational burden,” “IFN-γ signature,” “T cell-inflamed signature,” “ctDNA,” “molecular residual disease,” “microbiome,” “oxidative stress,” “ferroptosis,” “PFDN2,” “CD64,” and “FCGR1A.”

We prioritized English-language articles that provided mechanistic, translational, or clinical evidence relevant to inflammation-driven tumor initiation, immune reprogramming, biomarker development, and immunotherapy response in HNSCC. Particular emphasis was placed on high-impact studies published between 2022 and 2025, pivotal clinical trials, clinical practice guidelines, single-cell and spatial profiling studies, and primary experimental studies supporting causal mechanisms. Earlier landmark studies were included when they established foundational concepts, such as cancer immunoediting, cancer immunity cycles, macrophage biology, ICB, or canonical inflammatory signaling. Studies were excluded if they were not focused on HNSCC or closely related head and neck cancer subsites, lacked direct relevance to inflammation, immune regulation, biomarkers, or immunotherapy, were not available in English, or provided only duplicate or non-peer-reviewed information. Because this review is narrative in scope, formal quantitative synthesis, risk-of-bias scoring, and meta-analysis were not performed.

## 3 Chronic inflammation as a driver of HNSCC initiation and progression

Head and neck squamous cell carcinoma (HNSCC) remains a globally prevalent malignancy with substantial incidence and mortality, and its clinical burden has persisted despite progress in diagnostics and multimodal therapy (1, 2). Across subsites (oral cavity, oropharynx, larynx, hypopharynx), carcinogenesis frequently emerges from a chronically perturbed mucosa in which inflammatory signals are continuously renewed rather than extinguished (1, 3). Within this setting, inflammation functions less as a transient “alarm” and more as an enabling infrastructure that amplifies oncogenic selection, tissue remodeling, and immune deviation over time (3, 22, 25). At a conceptual level, the inflamed mucosa behaves like a permissive niche where stromal cells, innate immune cells, and epithelium become mutually reinforcing components of a pro-tumor ecosystem (1, 30, 31). This ecological perspective is clinically meaningful because HNSCC can arise through carcinogen-driven genomic alteration or virus-associated malignant transformation, yet both routes can converge on persistent inflammatory and immune-evasive states (1, 3, 32, 33).

The etiologic dichotomy between HPV-positive and HPV-negative disease provides a natural lens to study inflammation-cancer coupling, because these tumors can differ in patient demographics, anatomic predilection, and immune composition (1, 3, 34). HPV-positive tumors often occur in younger non-smokers and display distinct immune landscapes and survival patterns compared with HPV-negative, carcinogen-associated tumors, indicating that upstream inflammatory triggers shape downstream immunity (1, 3, 34). Conversely, smoking-related tumors show characteristic genomic alterations and copy number events that coexist with chronic inflammatory exposure, illustrating how mutational architecture and inflammation co-develop (1, 3, 32). These contrasts highlight that “inflammation” is not a monolith: viral persistence, carcinogen injury, and dysbiosis can each create partially overlapping yet biologically distinct inflammatory programs (1, 3, 12, 35).

Mechanistically, early tumorigenesis can be driven by the capacity of inflammatory signaling to stabilize survival pathways and to bias the immune context toward tolerance rather than clearance (22, 25, 36). STAT3/STAT5 activation in HNSCC is repeatedly described as supporting survival and proliferation, while simultaneously shaping an immune-permissive environment through inflammatory cross-talk (1, 3, 36). In parallel, oncogenic signaling can elevate checkpoint ligand availability– particularly PD-L1– thereby converting inflammatory cues into functional suppression of tumor-reactive T cells (1, 22, 37). RAS signaling is one instructive example, as oncogenic RAS can drive inflammatory cytokine outputs and increase tumor-cell PD-L1 expression, linking oncogenic metabolism and inflammation to immune escape (1, 3, 22). This is consistent with a broader principle that tumor cells can actively “engineer” inflammation, rather than merely adapting to it, by producing factors that recruit or educate suppressive myeloid populations (16, 30, 31, 37). The major inflammation-linked signaling pathways, their upstream regulators, downstream phenotypes, and candidate biomarkers are summarized in **Table 1**.

**Table 1:** Inflammation-linked signaling pathways and candidate biomarkers in HNSCC.

Pathway/axis	Common upstream activators in HNSCC	Key regulatory nodes (core hubs)	Dominant tumor function	Induced cellular phenotypes	Immune/TME consequences	Recommended biomarkers/readouts for IHC/IF/RNA profiling
NF-κB pathway	Survival Inflammatory amplification	IKK complex (IKKα/IKKβ/NEMO), p65/RelA, IκBα degradation	Survival Inflammatory amplification	Inflammatory state Stress adaptation EMT	Myeloid recruitment Immune suppression	p-p65 (nuclear), IκBα loss, COX-2 (PTGS2), IL-6, TNF-α, CXCL8/IL-8; PD-L1 as a context-dependent immune-evasion readout
STAT3/STAT5 signaling (JAK-STAT)	Proliferation Survival Immune tolerance	IL-6R/gp130, JAK1/2, pSTAT3 (Y705), STAT5	Proliferation Survival Stemness	Proliferation; Survival; Stemness; Immune tolerance	Immune tolerance Checkpoint link	pSTAT3, pSTAT5, IL-6, SOCS3; PD-L1 as a downstream immune-evasion marker
IL-6 / TNF-α inflammatory axis	Inflammatory reinforcement Therapy resistance	IL-6–JAK–STAT3; TNFR–IKK–NF-κB cross-activation	Inflammatory reinforcement Therapy resistance	Immune tolerance Stress adaptation, EMT priming	Suppressive milieu Poor ICB response	IL-6, TNF-α (tissue/serum), pSTAT3, p-p65; PD-L1 as a downstream convergence marker
TGF-β signaling	Invasion; Metastasis; Stromal remodeling	TGFβR1/2, SMAD2/3, SMAD4 status	Invasion Metastasis	EMT Motility Fibrosis Immune tolerance	T-cell suppression Tolerogenic niches	pSMAD2/3, Zeb1, Vimentin, N-cadherin, Snail/Slug; collagen/ECM markers (COL1A1), α-SMA (CAF)
PI3K–mTOR–4EBP1–SOX2 axis	Survival Stemness Resistance	PI3K, AKT, mTORC1, p-4EBP1, translational control; SOX2	Stemness	Stemness Plasticity Resistance	Immune escape Therapy resistance	pAKT, p-mTOR, p-4EBP1, SOX2, ALDH1A1, CD44 variant isoforms including CD44v
RAS / MAPK signaling (RAS–RAF–MEK–ERK)	Proliferation Survival Cytokine output	RAS, RAF, MEK, pERK	Proliferation Survival Cytokine output	Growth advantage Immune evasion	PD-L1 upregulation Immune escape	pERK, RAS/MAPK activation signatures; IL-6/IL-8; PD-L1
HA–CD44 adhesion/ ECM axis	Invasion; Migration; Stemness	CD44 isoforms, HA synthases (HAS), downstream cytoskeletal adapters (ERM)	Invasion Migration	EMT-like motility CSC maintenance	Niche formation Myeloid crosstalk	CD44 variant isoforms, including CD44v; HA content; HAS2; MMPs; EMT markers, including ZEB1 and Vimentin; CSC-associated markers, including ALDH1A1 and SOX2
Oxidative-stress adaptation (NRF2/HO-1; COX-2)	Stress tolerance Survival Resistance	NRF2-KEAP1, HO-1, COX-2	Stress tolerance Resistance	Stress-adapted aggressive state	Immunosuppressive tone	Nuclear NRF2, HO-1, COX-2, HSPs, metallothioneins

* Selected biomarkers represent practical readouts for IHC/IF, bulk RNA, or spatial/single-cell profiling. PD-L1 and EMT/CSC markers should be interpreted according to cellular source and microenvironmental context.

This table summarizes key signaling axes (e.g., NF-κB, JAK-STAT) connecting chronic inflammation to tumor progression and immune escape. It details upstream activators, resulting phenotypes, and practical biomarkers for assessing pathway activity in clinical or experimental settings.

Microbial and dysbiosis-related inflammation adds another upstream route into this early ecosystem (1, 12, 35). Intratumoral bacteria can influence initiation and progression by altering tumor-cell biology, immune responses, and the TME. In OSCC-related contexts, dysbiosis enriched for Fusobacterium nucleatum and Porphyromonas gingivalis can act as a chronic inflammatory trigger that supports epithelial–mesenchymal transition (EMT) and immune escape (1, 12, 35). Such host–microbe interactions plausibly contribute to a “primed” inflammatory state in which innate sensing is repeatedly engaged, macrophage polarization is skewed, and epithelial plasticity is promoted (1, 12, 25, 30). From a translational viewpoint, these observations suggest that microbial ecology may influence early immune editing and later therapy sensitivity by shaping baseline inflammatory tone (1, 34, 35).

A second foundational axis is oxidative stress and stress-response adaptation (1, 38, 39). Multiple inflammation- and oxidative-stress–linked molecules (e.g., heat shock proteins, metallothioneins, NRF2/HO-1 signaling, COX-2, and regulatory micro-RNAs) are pathologically relevant in head and neck and laryngeal squamous malignancies (1, 38, 39). Such stress-response programs can buffer malignant cells against hostile conditions created by chronic inflammation, thereby allowing mutated clones to persist and expand under selective pressure (1, 3, 39). COX-2–related inflammatory enzyme activity is similarly positioned as a plausible pathological driver that can reinforce tumor-promoting inflammation and suppress effective anti-tumor immunity (1, 18, 38). Together, these adaptations imply that early carcinogenesis is not solely a mutational story but also a story of how inflammatory stress is metabolically and transcriptionally “absorbed” by emerging malignant populations (1, 3, 40).

Inflammation also interfaces with stemness and invasion programs through extracellular-matrix (ECM) and adhesion signaling (1, 29, 41, 42). ECM reprogramming contributes to tumor development by altering macromolecular composition, degradative enzyme activity, and tissue stiffness, thereby redirecting cell fate and immune trafficking (1, 41, 43). Hyaluronic acid (HA)–CD44 engagement links growth- and invasion-promoting pathways, with HA–CD44 binding promoting migration and invasion while also enhancing stemness programs (1, 29, 42). Because CD44 is also a cancer stem cell (CSC)-associated marker in HNSCC and a mediator in the TME, HA–CD44 signaling becomes a plausible conduit through which inflammation and myeloid education can reinforce CSC-like states (1, 30, 37, 42). In this view, the inflamed microenvironment does not simply “support” tumor cells; it can actively tune epithelial identity toward plastic, therapy-resistant phenotypes that are more capable of immune escape (1, 3, 22, 42).

Finally, in HPV-associated disease, host genomic alterations linked to viral integration can directly modulate immune-evasion pathways (1, 3, 33). Recurrent HPV integration hot spots include sites such as CD274, and the hypothesis is advanced that host genomic alterations associated with HPV integration are critical contributors to the pathogenesis of many HPV-positive primary cancers (1, 3, 33). Because CD274 encodes PD-L1, these integration-associated events provide a direct mechanistic bridge between viral oncogenesis and checkpoint ligand up-regulation (1, 22, 33). Thus, across carcinogen-driven, microbe-perturbed, and virally driven routes, early inflammation can converge on shared downstream themes: stress adaptation, myeloid skewing, ECM reprogramming, and checkpoint reinforcement (1, 12, 35, 42). These convergent circuits plausibly pre-configure later immunotherapy responses by establishing suppressive niches and selecting for clones optimized for immune avoidance (1, 34, 37, 44).

To integrate these pathways across disease evolution, a temporal and causal framework can be proposed. During the initiating mucosal-injury stage, tobacco/alcohol-associated oxidative stress, HPV persistence, and dysbiosis predominantly activate epithelial NF-κB signaling and cytokine circuits such as IL-6 and TNF-α, thereby recruiting and educating innate immune cells. During premalignant lesion formation, NF-κB and STAT3 can become mutually reinforcing through inflammatory cytokine feedback, supporting epithelial survival, proliferation, and immune tolerance. At this stage, TGF-β may remain context-dependent, retaining growth-restraining effects in some epithelial compartments while progressively promoting stromal reprogramming, immune deviation, and epithelial plasticity as lesions advance.

**Figure 1:**
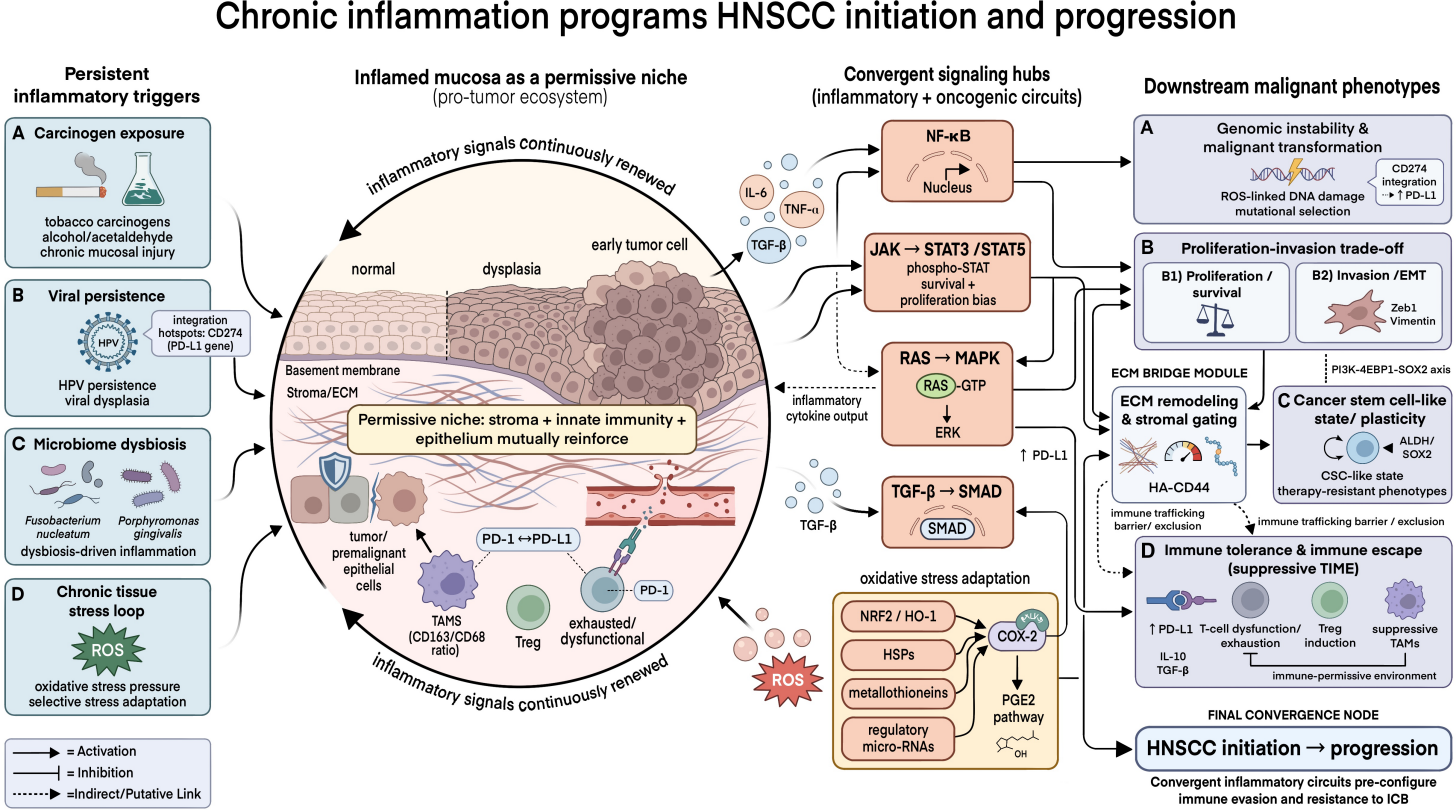
Chronic inflammation programs HNSCC initiation and progression. The schematic illustrates how persistent inflammatory triggers shape head and neck squamous cell carcinoma (HNSCC) initiation, progression, immune evasion, and resistance to immune checkpoint blockade (ICB). Carcinogen exposure, viral persistence, microbiome dysbiosis, and chronic oxidative or tissue stress converge on an inflamed mucosal niche composed of epithelial, stromal, and immune components. Continuously renewed inflammatory signals activate convergent signaling hubs, including NF-κB, JAK–STAT3/STAT5, RAS–MAPK, TGF-β–SMAD, and NRF2/HO-1 pathways. These interconnected circuits promote genomic instability, proliferation–invasion trade-offs, extracellular matrix (ECM) remodeling and stromal gating, cancer stem cell-like plasticity, and an immunosuppressive tumor immune microenvironment (TIME). Together, these processes drive malignant transformation, tumor progression, immune escape, and ICB resistance. Solid arrows indicate activation, blunt-ended lines indicate inhibition, and dashed arrows indicate indirect or putative links.

During invasive progression, TGF-β increasingly cooperates with NF-κB/STAT3-linked myeloid and CAF programs to promote EMT-like plasticity, extracellular matrix reprogramming, immune exclusion, and metastatic competence. In treatment-exposed or immunotherapy-resistant tumors, these circuits may shift from tumor-promoting inflammation to a therapy-shielding ecology: NF-κB/STAT3 signaling can sustain checkpoint ligand expression, cytokine feedback, and myeloid recruitment, whereas TGF-β can limit T-cell penetration and stabilize CAF-mediated stromal barriers. Therefore, NF-κB, STAT3, and TGF-β should be interpreted not as isolated static pathways but as stage-specific and compartment-specific regulators whose functions evolve during HNSCC progression and treatment resistance (**Figure 1**).

## 4 Inflammatory reprogramming of the TME and immune escape

The HNSCC tumor microenvironment (TME) functions as a dynamic immune ecosystem rather than a passive background during malignant transition (1, 30, 31). Macrophages are central to this ecosystem, but their effects are context-dependent: they can either restrain malignant growth or support immune escape and progression (1, 12, 30, 31). This plasticity reflects the physiological role of myelomonocytic cells in host defense, tissue damage responses, and repair (1, 12, 25). Within tumors, macrophages can become immunosuppressive, preventing tumor cell attack by natural killer (NK) and T cells during progression and after chemo- or immunotherapy (1, 31, 37). Clinically, TAM abundance is often associated with poor prognosis, and preclinical studies identify pathways that regulate TAM infiltration and polarization during tumor progression (1, 16, 45).

From the standpoint of immunotherapy, suppressive myeloid programs matter because checkpoint blockade targets are embedded within broader immunosuppressive circuits (1, 37, 44). Myelomonocytic cells are key components of the suppressive circuits that limit ICB efficacy, suggesting that response depends on both T-cell reinvigoration and myeloid context (1, 37, 44). Recent evidence further supports the importance of monocyte-specific inflammatory programs in HNSCC. Feng et al. identified plasma PFDN2 as a potential protective systemic regulator that suppresses HNSC progression by restricting CD64/FCGR1A-positive monocyte-driven inflammatory microenvironments. By integrating Mendelian randomization, plasma proteomics, immune-cell phenotype analysis, single-cell sequencing, virtual gene knockout, molecular docking and kinetic simulation, and HNSC tissue validation, this study linked systemic plasma protein regulation to a local monocyte-associated inflammatory niche. Importantly, the PFDN2–CD64/FCGR1A axis suggests that monocyte-driven inflammation is not only a descriptive feature of the HNSCC microenvironment but may represent a modifiable upstream regulator of immune suppression and tumor progression (46).

Consequently, the same checkpoint ligand (PD-L1) may carry different implications depending on whether it is expressed predominantly by tumor cells or by TAMs and other myeloid cells (1, 2, 37). This source-dependence motivates caution when interpreting PD-L1 readouts and supports the need for multi-compartment immune profiling rather than tumor-cell-restricted assays (1, 37, 44).

Inflammation drives this reprogramming through both soluble mediators and physical architecture (1, 29, 41). ECM alterations– composition, stiffness, and degradative dynamics– being under cellular control and capable of shaping tumor development, invasion, and immune behavior (1, 41, 43). Cancer-associated fibroblasts (CAFs) are heterogeneous TME components that support invasion, immune exclusion, and therapy resistance through multiple pathways (1, 29, 40). In HNSCC, tumor-derived signals such as basic fibroblast growth factor (bFGF) can modulate CAFs through FGFR engagement, illustrating how malignant epithelium actively instructs stromal programs (1, 29, 40). This stromal instruction is immunologically consequential because CAF-driven reprogramming can generate immune-excluded micro-anatomies and sustain cytokine gradients that blunt effective lymphocyte infiltration (1, 29, 43).

Chemokine programs add another layer of inflammatory control over immune composition (1, 37, 47). Chemokine signaling is explicitly proposed as a mechanism of resistance to checkpoint inhibitors, underscoring that immune cell migration and extra-vasation can be rate-limiting for therapy (1, 44, 47). The CXCL9/10/11–CXCR3 axis functions as an immune-trafficking module rather than a descriptive chemokine signature. Primary experimental studies have shown that intratumoral CXCR3 signaling, particularly CXCL9 derived from antigen-presenting myeloid cells or dendritic cells, is required for productive CD8^+^ T-cell responses after anti-PD-1 therapy. Similarly, macrophage-derived CXCL9 and CXCL10 have been shown to promote T-cell infiltration and antitumor immune responses following ICB (47–49). However, the same chemokine circuits can also be entangled with inflammatory reprogramming and may contribute to dysfunctional immune states if the surrounding TME enforces exhaustion or exclusion (1, 37, 47). Thus, chemokines should be interpreted as context-dependent signals whose net effect depends on stromal gating, myeloid suppression, and checkpoint activity (1, 29, 37, 43).

At the effector-cell level, chronic inflammation repeatedly fosters exhaustion-like programs (1, 34, 44). Despite the clinical success of PD-1/PD-L1 and CTLA-4 targeting, only a subset of patients show durable benefit, implying that T-cell dysfunction and/or failure of infiltration remain major bottlenecks (1, 2, 44). In this context, the tumor immune microenvironment (TIME) can appear lymphocyte-infiltrated yet still be functionally constrained, because exhausted or suppressed T cells do not execute effective cytotoxic programs (1, 37, 44). This is consistent with the broader theme that immunotherapy must consider “all key players” in the complex HNSCC TME to further enhance tumor-specific T-cell responses (1, 34, 37). Accordingly, the inflammatory TIME is best viewed as a network that couples T-cell states to myeloid programming, stromal architecture, and cytokine signaling (1, 29, 30, 37).

Therapeutic reprogramming of the TIME is therefore frequently discussed in terms of shifting macrophage states or interrupting myeloid checkpoints (1, 16, 50, 51). Blocking the CD47–SIRPα axis is presented as a strategy that improves immune responses to tumor cells by removing inhibitory signals from myeloid cells, thereby enhancing phagocytic and antigen-processing functions (1, 16, 50). Within the same conceptual space, CD103^+^ conventional type 1 dendritic cells (cDC1s) provide a mechanistic bridge between innate sensing and checkpoint responsiveness. Primary tumor-model studies have shown that CD103^+^ DCs can transport intact tumor antigens to draining lymph nodes, prime tumor-specific CD8^+^ T cells, and are required for optimal antitumor activity after PD-L1 blockade. These findings suggest that checkpoint therapy depends not only on T-cell reinvigoration but also on preserved antigen uptake, cross-presentation, and DC–T-cell crosstalk (52–54). These observations imply that effective checkpoint therapy requires not only “releasing the brake” on T cells but also maintaining robust antigen presentation and innate-adaptive coordination (1, 44, 50). Thus, inflammatory reprogramming of the TIME is both a driver of resistance and a map of actionable bottlenecks for combination design (1, 34, 37, 50).

**Figure 2:**
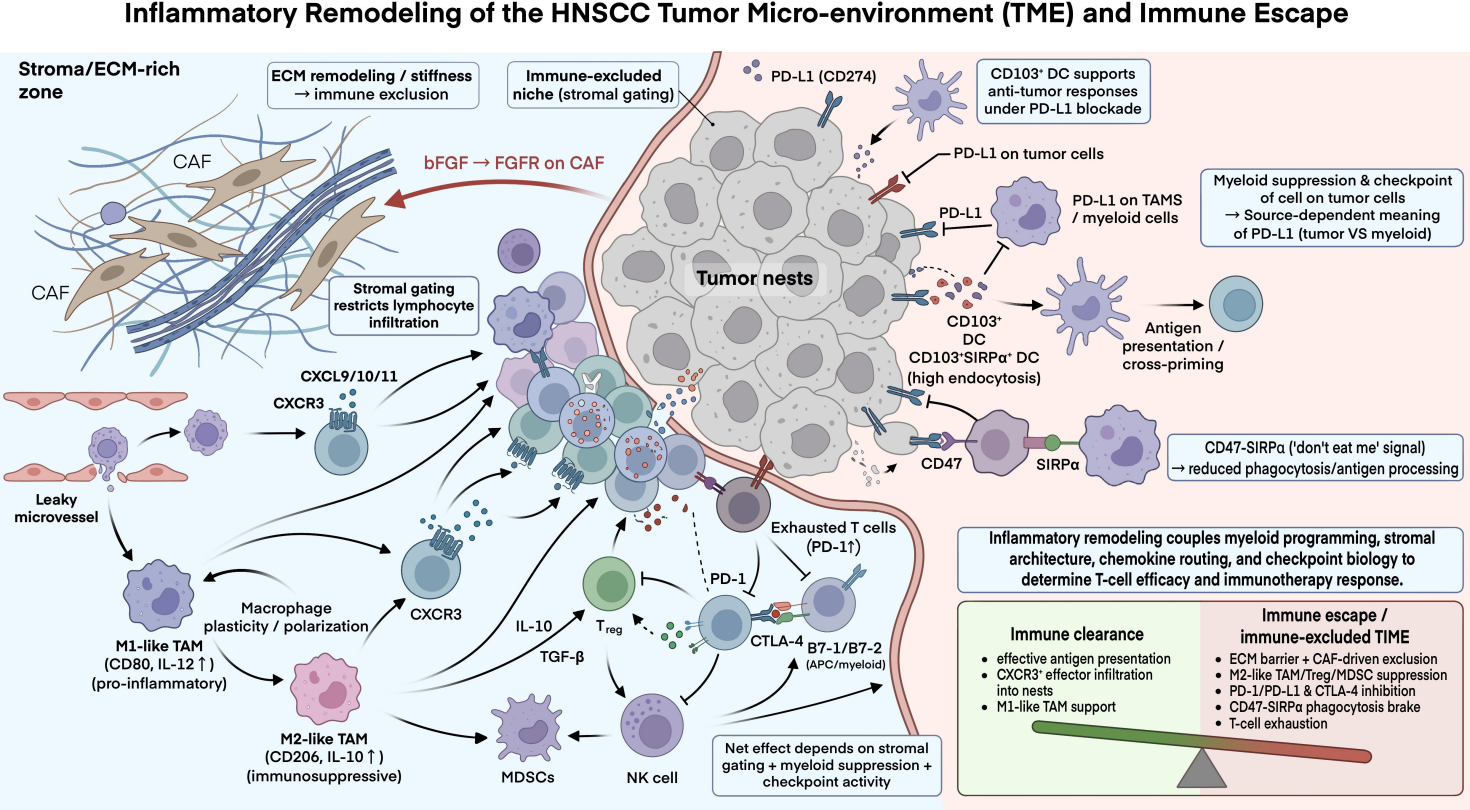
Inflammatory reprogramming of the HNSCC tumor microenvironment and immune escape. This schematic depicts the HNSCC microenvironment as an immune-excluded ecosystem. Tumor-instructed CAFs remodel the ECM to physically block T-cell infiltration (stromal gating). Concurrently, immunosuppressive M2-like TAMs and inhibitory checkpoints (PD-L1, CD47) suppress anti-tumor immunity. Despite chemokine recruitment and antigen presentation by CD103^+^ DCs, these structural and chemical barriers collectively drive T-cell exhaustion and immune evasion.

Finally, emerging modalities in the reference corpus emphasize that immune reprogramming can be pharmacodynamically shifted, providing tangible endpoints for translational work. For example, immunostimulatory interventions described in the text can increase activated T-cell fractions within tumors and alter CD4/CD8 composition, illustrating measurable TIME reconfiguration (1, 37, 55). Similarly, the development of engineered vesicle-based or nano-material strategies aims to stimulate innate/adaptive immunity while addressing toxicity constraints, highlighting the practical challenges of reprogramming the TIME (1, 55, 56). Taken together, the inflammatory TIME in HNSCC is best understood as a structured, multi-compartment system in which macrophage plasticity, stromal gating, chemokine routing, and antigen-presentation competence jointly determine immune efficacy (**Figure 2**) (1, 29, 50). This systems view explains why single-marker prediction often fails and why rational immunotherapy combinations increasingly target myeloid, stromal, and trafficking axes alongside PD-1/PD-L1 blockade (1, 34, 37, 47).

Despite recent breakthroughs, both primary and acquired resistance to immunotherapy remain formidable obstacles. Many HNSCC patients do not achieve lasting benefit, as inherent tumor escape mechanisms and adaptive immune evasion ultimately dampen treatment efficacy (1, 5). For example, the terminal exhaustion of tumor-specific T cells can drastically reduce the effectiveness of PD-1/PD-L1 blockade, and immunosuppressive cells such as M2-polarized TAMs, regulatory T cells, and myeloid-derived suppressor cells (MDSCs) further enforce T-cell dysfunction and maintain a tolerant tumor milieu, thereby driving both initial resistance and eventual immune escape (1, 34). Impaired T-cell persistence and memory may also lead to tumor relapse and acquired resistance after an initial immunotherapy response.

## 5 Inflammation-linked biomarkers and single-cell and spatial profiling

Because only a subset of patients experience durable benefit from ICB, biomarker development is moving from simple tumor-cell measurements toward integrated representations of tumor-immune dynamics (1, 34, 44).

To improve clinical interpretability, inflammation-linked biomarkers in HNSCC can be organized into three translational tiers. The first tier includes clinically used or near-clinical biomarkers, such as PD-L1 combined positive score (CPS), HPV/p16 status, tumor mutational burden (TMB), peripheral inflammatory indices such as the neutrophil-to-lymphocyte ratio (NLR), and circulating tumor DNA or molecular residual disease (ctDNA/MRD) assays (57–59). These markers are closest to clinical decision-making because they can inform treatment eligibility, risk stratification, or post-treatment surveillance, although their predictive strength varies by treatment setting and assay platform.

The second tier includes biomarkers under active clinical translation, including IFN-γ or T cell-inflamed gene-expression signatures, spatial distribution of tumor-infiltrating lymphocytes (TILs), and TAM/CD8^+^ T-cell ratios (60–62). These readouts move beyond single-marker expression by capturing whether antitumor immunity is functionally active, spatially excluded, or myeloid suppressed (63).

The third tier includes exploratory biomarkers, such as LAMP3^+^ dendritic cell states, CD163/CD68 macrophage ratios, Zeb1, Vimentin, CD44 isoforms, microbiome-associated profiles, oxidative-stress and ferroptosis-related markers, and CAF/ECM descriptors. Within this exploratory-to-translational tier, plasma PFDN2 may be considered a systemic inflammatory regulator and convertible biomarker linking circulating proteomic information with monocyte-driven tumor ecology. The PFDN2–CD64/FCGR1A axis is particularly relevant because it connects a measurable plasma protein to a defined immune-cell phenotype, CD64-positive monocytes, and to HNSC tissue-level validation. Unlike purely tissue-restricted markers, such a plasma protein–immune cell axis may provide a bridge between non-invasive risk assessment, immune microenvironment interpretation, and therapeutic hypothesis generation. However, its clinical application remains exploratory and requires independent cohort validation, standardized assay development, and prospective testing to determine whether PFDN2 adds predictive value beyond established biomarkers such as PD-L1 CPS, HPV/p16 status, TMB, NLR, and ctDNA/MRD (46). These markers are biologically informative but require further analytical standardization, prospective validation, and demonstration of incremental clinical utility before routine application.

Within the first tier, PD-L1 immunohistochemistry remains the most widely used immunotherapy-related biomarker in HNSCC, and lymphocyte infiltration remains an important correlate of tumor immune activity (1, 2, 44). However, clinical interpretation should emphasize PD-L1 CPS rather than tumor proportion score (TPS) alone because PD-L1 CPS incorporates staining in both tumor cells and tumor-associated immune cells (64, 65). HPV/p16 status should also be considered a clinically established stratification factor because it defines a biologically and prognostically distinct subgroup, particularly in oropharyngeal squamous cell carcinoma (64, 66, 67). By contrast, TMB, NLR, and ctDNA/MRD are better regarded as clinically relevant or near-clinical adjuncts rather than standalone predictors, as their utility depends on assay standardization, disease setting, and prospective validation (60).

PD-L1 is emphasized as a central immune-evasion molecule and a direct therapeutic target, making it an intuitive candidate for predicting response to PD-1/PD-L1 inhibitors (1, 22, 44). At the same time, PD-L1 is regulated by inflammatory signaling and oncogenic pathways, meaning its expression is dynamic and can vary by region, time, and cellular source (22, 37). Therefore, PD-L1 measurement is informative but insufficient as a standalone predictor (1, 2, 44).

A key limitation concerns cellular source (1, 2). Tumor-cell PD-L1 expression may reflect tumor-intrinsic oncogenic signaling or adaptive immune resistance driven by local IFN-γ exposure, whereas immune-cell or myeloid-cell PD-L1 may instead reflect an inflamed but suppressive microenvironment (68). Myelomonocytic cells and macrophages are key contributors to immunosuppressive pathways targeted by checkpoint blockade, and PD-L1 expression on these populations can represent inflammatory tone rather than tumor antigenicity (16, 30, 31, 37). Accordingly, a PD-L1 CPS-positive tumor may reflect biologically distinct states: tumor-cell-dominant PD-L1 expression, immune-cell-dominant PD-L1 expression, or a mixed pattern (64, 65). These states may have different implications for antigenicity, myeloid suppression, and response to PD-1/PD-L1 blockade, supporting the need to interpret PD-L1 together with myeloid context, stromal architecture, and effector T-cell competence (1, 29, 37, 43, 44).

Macrophages and other myeloid–lineage cells can express a range of immune checkpoint ligands– including PD-L1, PD-L2, B7-H4, and the B7-1/B7–2 ligands that engage CTLA-4– especially under the influence of inflammatory cytokines or within hypoxic tumor regions (12, 37). In certain malignancies (such as hepatocellular carcinoma, glioblastoma, and pancreatic cancer), TAMs indeed display high levels of PD-L1 and/or B7-H4, although it remains unclear to what extent these macrophage-derived “myeloid checkpoint” signals contribute to overall immune suppression in the TME (37). Therefore, the use of PD-L1 immunohistochemistry as a predictive biomarker in HNSCC requires careful consideration of the cellular source of PD-L1. In particular, PD-L1 positivity driven predominantly by abundant TAMs may confound the correlation between PD-L1 status and immunotherapy outcomes, warranting caution in interpretation and underscoring the importance of context-specific biomarker analysis (1, 2, 37).

A second limitation is spatial and temporal heterogeneity (69–71). PD-L1 expression may vary across tumor center, invasive margin, stromal regions, recurrent lesions, and metastatic sites; therefore, a single biopsy may misclassify the immune state of a heterogeneous tumor. Intratumoral heterogeneity can reduce the reliability of both CPS and TPS when they are derived from limited tissue, particularly if immune cells are spatially concentrated at the invasive margin or in stromal compartments rather than evenly distributed within tumor nests (69, 70). Longitudinal changes after radiotherapy, chemotherapy, or immunotherapy may further alter PD-L1 status and immune-related gene-expression surrogates (71). Therefore, PD-L1 CPS should be interpreted as a useful but spatially constrained clinical biomarker, ideally integrated with TIL distribution, TAM/CD8^+^ ratios, stromal exclusion patterns, and, when feasible, multi-region or spatially resolved profiling (3, 61, 63).

Inflammation-associated biomarkers extend far beyond PD-L1, spanning stress-response proteins, inflammatory enzymes, micro-RNAs, and metabolic vulnerabilities (1, 38, 39, 72). In laryngeal and head and neck squamous malignancies, biomarkers correlated with oxidative stress and inflammation include HSPs, metallothioneins, NRF2, HO-1, COX-2, and micro-RNAs, reflecting persistent inflammatory and environmental stress exposure. These markers are attractive because they index the “adaptation layer” of tumor cells under chronic inflammation, which can shape aggressiveness and therapeutic sensitivity (39, 73). COX-2 and inflammatory prostaglandin pathways are positioned as tumor-promoting and immunosuppressive, suggesting that their measurement may reflect a pro-tumor inflammatory state. Similarly, NRF2-centered antioxidant programs can indicate the degree of oxidative buffering, which may influence response to radiotherapy, chemotherapy, and potentially immune-mediated killing (1, 38, 72, 73).

Metabolic biomarkers are increasingly relevant to inflammation-linked HNSCC biology, especially in OSCC. Recent OSCC studies have shown that NRF2-centered antioxidant programs can potentiate radioresistance through metabolic modulation, indicating that redox buffering is not only a passive stress-response state but also a therapy-resistance mechanism. In parallel, ferroptosis-related studies have demonstrated that OSCC cells rely on anti-ferroptotic defenses such as GPX4 and SLC7A11 to survive lipid-peroxidation pressure. For example, modulation of the p53/SLC7A11 axis can enhance ferroptosis and suppress malignant behaviors in OSCC cells, while broader multi-omics analyses have further linked intercellular communication within the OSCC microenvironment to oxidative-stress programs that promote tumor progression. Therefore, oxidative-stress and ferroptosis-related markers, including NRF2/HO-1, GPX4, SLC7A11, and lipid-peroxidation-associated pathways, may serve as functional readouts of inflammatory stress load and as candidate biomarkers for predicting sensitivity to radiotherapy, chemotherapy, and ferroptosis-inducing or immune-combination strategies (68, 74–77). Thus, inflammation-linked metabolism provides a tractable biomarker–target interface that complements purely immune-centric markers (1, 44, 72, 77).

Recent mechanistic evidence further connects ferroptosis-related stress responses with innate immune activation. Li et al. showed that reciprocal regulation between ferroptosis and the STING–type I interferon pathway can suppress HNSCC growth through dendritic cell maturation, suggesting that ferroptosis-related biomarkers may also reflect an immune-activating metabolic state rather than only tumor-intrinsic oxidative vulnerability (77).

Stromal and ECM-associated markers also function as inflammation-linked biomarkers because they encode the “routing rules” for immune infiltration (29, 41, 43). CAFs are described as essential and heterogeneous TME components that can promote tumor progression, and HNSCC-derived cues, such as bFGF, can redirect CAF behavior (1, 29, 40). Because CAF-rich or ECM-stiff tumors can restrict immune access, stromal markers can identify immune-excluded phenotypes that may fail checkpoint therapy despite antigenicity (1, 29, 43). ECM reprogramming is described as a controlled process involving macro-molecular composition, degradative enzymes, and stiffness, and these parameters can act as functional biomarkers of immune permissiveness (41, 43). Accordingly, integrated biomarker panels that combine PD-L1/TIL readouts with stromal and ECM descriptors are more likely to represent the functional immune state of HNSCC (1, 29, 43, 44).

Finally, “cellular-function” biomarkers that index antigen presentation and innate-adaptive coupling are increasingly important. The contribution of CD103^+^ DCs to anti-tumor responses induced by PD-L1 blockade, and the high endocytic capacity of CD103^+^ SIRPα^+^ DCs, implies that DC competence is a biomarker of effective checkpoint response (1, 37, 50). Similarly, myeloid-checkpoint biology such as the CD47-SIRPα axis can serve as both a therapeutic target and a functional biomarker of whether macrophage-mediated clearance is inhibited (1, 16, 50).

Taken together, inflammation-linked biomarkers in HNSCC should not be interpreted as an unranked catalogue. A clinically useful framework should first prioritize markers with established or near-clinical applicability, such as PD-L1 CPS and HPV/p16 status (64–67), then integrate translational immune-activity readouts such as IFN-γ/T cell-inflamed signatures, TIL spatial distribution, and TAM/CD8^+^ ratios (60) (61, 63), and finally position exploratory markers—including LAMP3, CD163/CD68, Zeb1, Vimentin, CD44, microbiome profiles, oxidative-stress markers, ferroptosis-related molecules, and CAF/ECM descriptors—as hypothesis-generating tools for mechanism discovery and patient stratification (12, 29, 38, 42, 72). This hierarchy clarifies that durable immunotherapy benefit is determined by multi-layer ecology, but also that only biomarkers with standardized assays, prospective validation, and added predictive value should be advanced toward routine clinical decision-making (1, 37, 44, 65).

Advanced detection strategies are increasingly needed because conventional pathology readouts cannot fully capture the spatial, cellular, and functional heterogeneity of inflammation-driven tumor ecosystems (1, 34). Single-cell and spatial profiling resolve immune and stromal sub-populations, enable inference of differentiation trajectories, and identify tissue neighborhoods that constrain or enable effective immunity (78). This is especially relevant in HNSCC, where distinct etiologies (HPV-positive vs HPV-negative) create different TMEs while still sharing certain convergent immune dysfunction patterns (3, 34). single-cell and spatial profiling therefore helps reconcile etiologic differences with shared therapeutic bottlenecks by identifying which cell states and interactions are common versus context-specific (34). In practice, these technologies provide a map for designing and selecting combination therapies that target rate-limiting nodes rather than superficial correlates. The principal high-dimensional single-cell and spatial profiling platforms, together with their readouts, spatial resolution, sample requirements, applications, strengths, and limitations, are summarized in **Table 2** (1, 34, 50).

**Table 2:** Overview of high-dimensional single-cell and spatial platforms for HNSCC ecosystems.

High-dimensional single-cell and spatial omics platforms for mapping inflammatory tumor ecosystems in HNSCC								
Comparative overview of outputs, resolution, biological insights, and applications in HNSCC immuno-oncology								
Primary analytical focus	Platform / technology	Primary readout	Spatial resolution	Throughput & sample type	Biological insights provided	Key HNSCC applications	Key strengths	Key limitations / pitfalls
Cell-state profiling	scRNA-seq	Transcriptome	None	High throughput; fresh dissociated tissue required	Cell types, rare states; activation signatures; cytokine programs	Immune/stromal atlases; TAM profiling; HPV-positive and HPV-negative landscapes; pathway scoring	Unbiased discovery; high dimensionality	Dissociation bias; no spatial context; dropouts; loss of fragile cells
Spatial distribution	snRNA-seq	Nuclear RNA	None	Compatible with frozen or archival samples; reduced dissociation-induced stress	Epithelial/stromal states; complementary to scRNA-seq	Fibrotic tumors; CAF programs; stress-response states	Broad sample compatibility	Lower cytoplasmic signal; no spatial context
Cell–cell interaction	CITE-seq	RNA and protein tags	None	Medium-to-high throughput; fresh samples; requires an antibody panel	Transcriptome and surface phenotype; improved state definition	Refining T-cell states; myeloid subsets; checkpoint co-expression	Links RNA and protein readouts; improved annotation	Panel size limits; antibody validation needed
Trajectory analysis	CyTOF / Spectral Flow	Protein markers, including surface and intracellular markers, 30–60+ markers	None	High throughput; fresh suspension	Lineages and functional signaling, including pSTAT and pNF-κB signaling	Quantifying composition; TAM polarization; therapy monitoring	Deep phenotyping; functional signaling detection	No spatial context; complex panel design and analysis
	Multiplex immunofluorescence (mIF)	Proteins (5-10+ markers)	Single-cell (segmentation dependent)	FFPE compatible; moderate throughput	Cell identities and spatial proximity; PD-L1 sourcing	Excluded versus inflamed phenotypes; immune neighborhoods; PD-L1 patterns	Clinically translatable; preserves histology	Lower multiplexing capacity than mass spectrometry-based imaging; segmentation errors and signal crosstalk
Spatial neighborhoods	IMC / MIBI-TOF	Protein (30-50+ markers)	~1 μm (subcellular)	FFPE; small regions of interest (ROIs); slow acquisition	High-plex spatial states; immune niches; interaction maps	CAF barriers; immunosuppressive synapses; tertiary lymphoid structures (TLS); spatial checkpoint biology	High multiplexing capacity and spatial fidelity	Small imageable area; high cost; complex analysis
	Spatial transcriptomics, such as Visium	RNA profiles from mixed-cell spots	Spot-level resolution rather than single-cell resolution	FFPE/Frozen; whole-section mapping	Regional programs; tissue gradients; neighborhood signatures	Exclusion borders; cytokine niches; pathway mapping, including TGF-β-associated zones	Whole-slide coverage; histology integration	Mixed-cell spots requiring computational deconvolution; variable sensitivity
High-resolution spatial transcriptomics	High-resolution spatial RNA profiling, such as Xenium or CosMx	Targeted RNA panels covering hundreds to thousands of genes	Single-cell to subcellular	Tissue sections; requires predefined panel	Precise identities and micro-niches; ligand–receptor adjacency	Tumor-immune contact zones; macrophage niches; localized PD-1/PD-L1 programs	Subcellular resolution; strong inference of spatial interactions	Limited to predefined gene panels; high cost; complex quality control
	ROI-based spatial proteomics	Protein in selected regions of interest (ROIs)	Region-of-interest/segment-level	FFPE; pathology-guided regions of interest (ROIs); moderate throughput	Compartment-level biology, including tumor and stromal regions of interest	Tumor–stroma comparison; compartment signatures; spatial biomarkers	FFPE-friendly; clinically adaptable design	Selection bias inherent to regions of interest; lower resolution than imaging
	scATAC-seq / multiome	Chromatin accessibility and RNA expression	None	Fresh nuclei required; complex computational analysis	Regulatory programs; lineage priming; TF activity	Drivers of tolerance/exhaustion; inflammation-induced regulatory shifts	Mechanistic insight into regulatory programs	Data sparsity; high cost; no spatial context
	Recommended use cases • Cell-type discovery: scRNA-seq and snRNA-seq • Functional phenotyping: CyTOF, spectral flow cytometry, and CITE-seq • Spatial neighborhoods: mIF, IMC/MIBI-TOF, and spatial transcriptomics • Micro-niche inference: high-resolution spatial RNA profiling combined with IMC/MIBI-TOF					Common analysis outputs • Cell-type atlases and state scores • Trajectory trees and neighborhood graphs • Cell–cell interaction networks • Source-aware PD-L1 mapping		

Resolution, multiplex capacity, and FFPE compatibility vary by specific platform and panel design. Interpretation requires rigorous quality control, including correction of batch effects, assessment of segmentation accuracy, and integration with pathological evaluation.

This table compares technologies for mapping immune and stromal landscapes, categorized by data modality (RNA/protein) and spatial resolution. It outlines sample requirements (fresh vs. FFPE) and biological outputs—ranging from cell phenotyping to niche analysis—alongside specific HNSCC applications and technical limitations.

Myeloid profiling is particularly informative because tumor-infiltrating myeloid cells (TIMs) are described as key regulators of tumor progression and as major determinants of immune suppression (16, 78). Pan-cancer analyses underscore that myeloid properties show both similarities and distinctions across tumor types, implying that general myeloid principles must be interpreted through tissue-specific inflammatory context (1, 30, 78). The references emphasize that monocytes and macrophages encompass multiple subsets, and macrophage polarization states (M1-like vs M2-like) capture only part of a broader functional continuum (1, 12, 30, 78). Given the central role of macrophages in HNSCC development, high-resolution myeloid mapping can reveal suppressive programs that are invisible to bulk IHC markers (1, 30, 31). This supports the rationale for TAM-targeted interventions and for biomarker panels that quantify myeloid state rather than myeloid presence alone (1, 16, 37).

Virus-associated head and neck tumors (e.g., nasopharyngeal carcinoma in the EBV setting) provide illustrative examples of what single-cell approaches can uncover. Single-cell analyses identify malignant and immune cell groups enriched for “viral carcinogenesis” and interferon-related pathways, showing how viral inflammation imprints transcriptional programs across compartments. Such signatures can be aligned with immune phenotypes (e.g., lymphoid enrichment or specific T-cell states), enabling mechanistic interpretation of why certain viral tumors exhibit distinct immune infiltration patterns (1, 3). These insights are directly relevant to HPV-positive HNSCC, where viral antigens, host genomic alterations, and checkpoint pathways intersect to shape immune behavior (1, 3, 33). Thus, single-cell and spatial profiling can unify virology, inflammation, and immune suppression into coherent, testable models of tumor behavior (1, 33).

Spatially resolved pathology complements single-cell omics by preserving tissue context (29, 41, 79). Because ECM composition and stiffness can be remodeled in cancer and are under cellular control, spatial methods can quantify where immune cells are blocked or guided by stromal architecture (1, 41, 43). Similarly, CAF heterogeneity and CAF–tumor signaling (e.g., bFGF–FGFR interactions) can be spatially mapped to understand immune exclusion niches (1, 29, 40). These data can explain why a tumor may appear “immune-infiltrated” in bulk but remains non-responsive if immune cells are confined to stromal borders rather than tumor nests. Accordingly, advanced spatial diagnostics can convert descriptive pathology into functional micro-anatomy that predicts therapy response (29, 41, 43).

High-resolution technologies are also valuable because they generate actionable pharmacodynamic endpoints (50, 55). For example, evidence that CD103^+^ DCs support PD-L1 blockade responses implies that monitoring DC abundance and endocytic state could serve as a pharmacodynamic marker during therapy (37, 50). Likewise, interventions that measurably shift tumor T-cell activation and CD4/CD8 composition demonstrate that TIME reprogramming is quantifiable and can be tracked as a treatment response signal. Nanomaterial and vesicle-based immunomodulatory approaches described in the references emphasize both potential and constraints, including the importance of toxicity and delivery considerations (1, 55, 56). Together, these approaches expand the diagnostic space from static markers (PD-L1, TILs) to dynamic systems readouts (myeloid state, antigen presentation, spatial routing, and functional activation) (34, 50).

In aggregate, single-cell and spatial profiling technologies enable the field to move from coarse classification to mechanistically grounded stratification (34, 78). They clarify how inflammation shapes tumor biology via stress-response adaptation, microbiome perturbations, myeloid plasticity, stromal gating, and checkpoint reinforcement (29, 35). They also provide a route to harmonize biomarkers with therapy design, pairing observed bottlenecks (e.g., deficient antigen presentation or myeloid checkpoint dominance) with rational combination interventions (34, 37, 50). For HNSCC, where etiologic diversity and microenvironment heterogeneity are major drivers of variable immunotherapy benefit, these methods provide the most direct path toward reproducible, evidence-linked precision immunotherapy (2, 3, 6). Thus, advanced detection strategies are not ancillary; they are central tools for decoding inflammation–cancer transition and for translating mechanistic insights into clinically actionable stratification frameworks (34, 43).

High-dimensional profiling technologies will be critical for guiding these next-generation therapies. Standardizing pathological biomarkers and integrating multiomics data (genomic, transcriptomic, proteomic, etc.) are crucial steps toward refining patient selection for individualized immunotherapy. For example, high-resolution single-cell and spatial profiling can resolve nuanced differences in immune infiltrates and inflammatory marker expression that are imperceptible to conventional pathology (78). Spatial mapping of immune biomarkers has already shown clinical promise: the tissue distribution pattern of PD-L1 expression, when considered alongside metrics such as the TAM-to-CD8^+^ T cell ratio, can distinguish responders from non-responders to immunotherapy in HNSCC more effectively than bulk measurements alone (11, 39, 62). Incorporating such spatially resolved and multi-parametric biomarkers with traditional clinical factors could enable more precise patient stratification and inform personalized combination treatment strategies (11, 39).

## 6 Targeting the inflammatory tumor ecosystem: therapeutic strategies and future outlook

Before discussing therapeutic combinations for established HNSCC, it is important to recognize that the inflammatory tumor ecosystem may already be shaped during early OSCC development and even at the precancerous stage. Single-cell and spatial analyses of oral mucosal precancerous lesions and very early-stage OSCC have revealed that altered epithelial states are accompanied by distinct fibroblast, monocytic, and regulatory T-cell subclusters, including an immune-inhibitory monocyte population and spatially organized VEGF-related signaling surrounding precancerous lesions. Recent single-cell and spatial work in lip cancer further indicates that neutrophil states are spatially and therapeutically plastic, including the emergence of anti-tumor neutrophil subsets after microwave thermochemotherapy (80). These findings suggest that myeloid reprogramming is not merely a late consequence of invasive cancer but may participate in disease initiation, immune suppression, and niche formation from early stages. These early myeloid alterations may also include MDSC-like suppressive states, suggesting that neutrophil/monocyte/MDSC reprogramming can begin before fully invasive disease and may precondition later immune exclusion and checkpoint resistance.

Consistently, single-cell studies of HNSCC have identified SPP1^+^ TAMs and MDSCs as abundant myeloid populations that interact with CD8^+^ T cells, endothelial cells, and other stromal compartments, thereby promoting immunosuppression, angiogenesis, and tumor progression. Therefore, early-stage OSCC provides a rational window for interventions aimed at preventing or reversing myeloid-driven inflammatory niche formation before immune escape becomes fully established (81–84).

Unfortunately, the overall response rates to immune checkpoint inhibitors in HNSCC remain disappointingly low in absolute terms, with only a minority of patients achieving meaningful tumor regression. This reality has driven the search for predictive biomarkers to better identify likely responders; however, single markers have proven inadequate, suggesting that multi-parameter combination approaches may be required to broaden the population that benefits from immunotherapy and potentially approach cure in this disease (1, 5). Accordingly, numerous ongoing trials are evaluating checkpoint inhibitors in combination with other treatment modalities-including radiotherapy, cytotoxic chemotherapy, and additional immunotherapies – with the goals of boosting response frequencies, prolonging the durability of remission, and even exploring the possibility of cures in advanced HNSCC. Given that many patients derive only limited benefit from current immunotherapies (and some experience significant immune-related toxicities), rational combination regimens have become a major focus of research (85). In the recurrent and metastatic setting, HNSCC remains a difficult-to-treat malignancy in which systemic therapy is often crucial. Immunotherapy is fundamentally aimed at restoring the functional anti-tumor capacity of the host immune system to counteract the tumor’s myriad immune evasion strategies. Broadly, immunotherapeutic approaches encompass tumor-specific monoclonal antibodies, therapeutic cancer vaccines, cytokine-based treatments, adoptive T-cell transfers, and other immune-modulating agents (6). Nevertheless, to further improve patient outcomes, novel immunotherapeutic strategies that simultaneously address multiple components of HNSCC’s complex TME are urgently needed-strategies that can more effectively amplify tumor-specific T cell responses and enhance overall treatment efficacy (34).

One such strategy is to blunt the tumor-promoting inflammation that fosters immune escape. Inhibiting key pro-inflammatory mediators (such as IL-6, TNF-α, or TGF-β) and their downstream signaling cascades can help alleviate immunosuppression within the tumor milieu. For example, blocking the IL-6– activated STAT3 pathway– a central driver of inflammation-induced immune tolerance– has been shown to down-regulate tumor cell PD-L1 expression and augment anti-tumor T cell responses (36). Early preclinical evidence further suggests that combining anti-inflammatory agents (e.g. IL-6 or TGF-β antagonists) with checkpoint blockade could synergistically enhance anti-cancer efficacy, providing a stronger cumulative relief of immune inhibition than either approach alone (36, 85).

Another complementary approach is to reprogram the TAMs that populate the HNSCC microenvironment. Suppressing the polarization of macrophages toward the tumor-promoting M2 phenotype– for instance, using inhibitors of CSF-1R or blocking “don’t-eat-me” signals like the CD47-SIRPα pathway– can relieve macrophage-mediated immunosuppression, thereby increasing macrophage-driven tumor cell clearance and improving responses to checkpoint inhibitors (12, 86). Conversely, actively driving TAMs toward a pro-inflammatory, M1-like state bolsters antigen presentation and T cell activation in the tumor context, effectively converting macrophages from accomplices of tumor growth into agents of tumor destruction (12, 86). Because TAMs play critical roles in tumor progression and immune evasion, shifting their phenotype to an M1 tumoricidal state is considered a promising therapeutic strategy. Notably, the FDA-approved nanoparticle ferumoxytol (a dextran-coated iron oxide formulation) was found to induce reactive oxygen species via a Fenton reaction, thereby repolarizing TAMs from an M2 toward an M1 phenotype and suppressing tumor progression and metastasis *in vivo*. In experimental models, ferumoxytol-treated macrophages exhibited significantly up-regulated M1 markers (e.g., CD80, IL-12) alongside down-regulated M2 markers (e.g., CD206, IL-10), confirming effective M2-to-M1 functional reprogramming and corresponding tumor growth inhibition (56).

Beyond immune cells, other stromal components can also be co-targeted to enhance immunotherapy. For example, mesenchymal stromal cells (MSCs) in the tumor stroma exert both pro- and anti-tumor influences, and transforming growth factor-β (TGF-β) isoforms (β1, β2, β3) regulate many facets of MSC biology and paracrine signaling impacting tissue regeneration, immune responses, and cancer progression. These observations reinforce that curbing inflammation-driven stromal crosstalk (in addition to reinvigorating T cells) may further tip the balance in favor of anti-tumor immunity.

Despite the clinical successes of antibodies against CTLA-4 and PD-1/PD-L1, only a subset of HNSCC patients experience durable responses, underscoring the need for a more comprehensive understanding of tumor-immune interactions (1, 5). The strength and timing of anti-tumor immune responses in any given individual are governed by a complex interplay of tumor-intrinsic, host, and environmental factors. Notably, the quality of intratumoral T-cell, natural killer (NK) cell, and even B-cell responses has been linked to immunotherapy outcomes, highlighting the multi-dimensional nature of this immune equilibrium (19). Investigators are now beginning to integrate such factors into predictive “immune profiles”; within the framework of the cancer– immunity cycle, these composite factors can be viewed as defining an innate immunological set-point unique to each patient (44). While current approved immunotherapies mainly target T-cell checkpoints, additional targets are on the horizon (51). Recent HNSCC-focused reviews of next-generation checkpoint strategies further highlight TIM-3, TIGIT, LAG-3, and VISTA as emerging targets that may complement PD-1/PD-L1 blockade, particularly in tumors with exhausted T-cell states and myeloid-dominant immune suppression. In particular, immunosuppressive myelomonocytic cells (for example, certain macrophage and myeloid subsets) constitute key elements of the immunosuppressive pathways that checkpoint blockade seeks to counteract, and they may offer avenues to better predict or potentiate responses to such therapies (34, 37).

In addition, an unexpected phenomenon termed “hyperprogressive disease” (HPD) has been observed in a subset of patients during anti-PD-1/PD-L1 therapy, wherein the tumor exhibits an accelerated growth rate following initiation of treatment. This deleterious response is associated with a markedly worse prognosis and is suspected to represent an immune-related adverse event triggered by checkpoint blockade (10). As the clinical use of PD-1/PD-L1 inhibitors continues to expand, pressing priorities now include identifying robust predictive biomarkers, unraveling mechanisms of resistance, understanding and mitigating hyper-progression, optimizing treatment sequencing and duration, managing immune-related toxicities, and refining clinical trial designs to better address these issues (87).

Beyond these immunotherapy-specific challenges, HNSCC remains biologically heterogeneous across anatomical subsites and etiological backgrounds (1, 4). This heterogeneity necessitates careful patient stratification and tailored therapeutic approaches (66). For example, HPV-positive oropharyngeal squamous cell carcinoma represents a distinct subgroup with favorable prognosis, and multiple clinical trials are examining treatment de-intensification strategies in this cohort to preserve survival outcomes while reducing treatment-related toxicity (87).

On the therapeutic front, however, a spectrum of novel immunotherapies is emerging to address these challenges. Cancer vaccines (including prophylactic HPV vaccines) and oncolytic virotherapies are being tested in HNSCC to provoke more robust anti-tumor immune responses. For instance, an engineered oncolytic herpesvirus encoding GM-CSF (talimogene laherparepvec) has demonstrated the capacity to induce vigorous tumor-specific immunity in early-phase trials. Adoptive cell transfer (ACT) approaches, such as T cell receptor (TCR)-engineered lymphocytes and chimeric antigen receptor (CAR) T cells, are also under active exploration; while ACT has achieved FDA approval in certain hematologic malignancies, its application in HNSCC remains mostly limited to Phase I clinical studies so far (1, 88).

Beyond T-cell-centric approaches, recent efforts have turned to harnessing innate immune mechanisms in HNSCC. Natural killer (NK) cells, for example, can recognize and eliminate malignant cells without MHC restriction, making NK-based therapies– alone or in combination with T cell-centric treatments– an attractive avenue (1, 19).

Pharmacological activation of innate immune pathways is also being explored. For example, an antibody–drug conjugate designed to agonize the STING pathway was shown to synergize with PD-L1 blockade. This strategy amplified dendritic-cell, T-cell, and NK-cell activation, promoted TAM repolarization toward an M1-like phenotype, and improved tumor control in preclinical models (55, 69, 77).

Clinically oriented evidence is also emerging: high cGAS-STING expression has been associated with improved efficacy of neoadjuvant chemo-immunotherapy in HNSCC, suggesting that STING-related signaling may serve not only as a therapeutic target but also as a context-dependent biomarker of innate immune activation. Another concept gaining traction is “trained innate immunity” (TII), wherein a brief stimulatory exposure to the innate immune system can reprogram myeloid cells (such as tumor-associated neutrophils) into long-lasting tumoricidal phenotypes; even a single TII intervention significantly suppressed tumor growth and conferred durable tumoricidal functionality in neutrophils in preclinical studies (1, 89).

In light of the innate immune system’s remarkable plasticity, investigators have also turned to macrophage-centered therapies as a novel treatment avenue (16, 25). One innovative approach involves engineering macrophages with chimeric antigen receptors (CARs) to confer tumor-targeting specificity. Early preclinical studies indicate that these CAR-modified macrophages (CAR-M) can efficiently infiltrate tumors, phagocytose cancer cells, and activate anti-tumor immune responses– representing a compelling strategy to complement T cell-centric immunotherapies (90).

**Figure 3:**
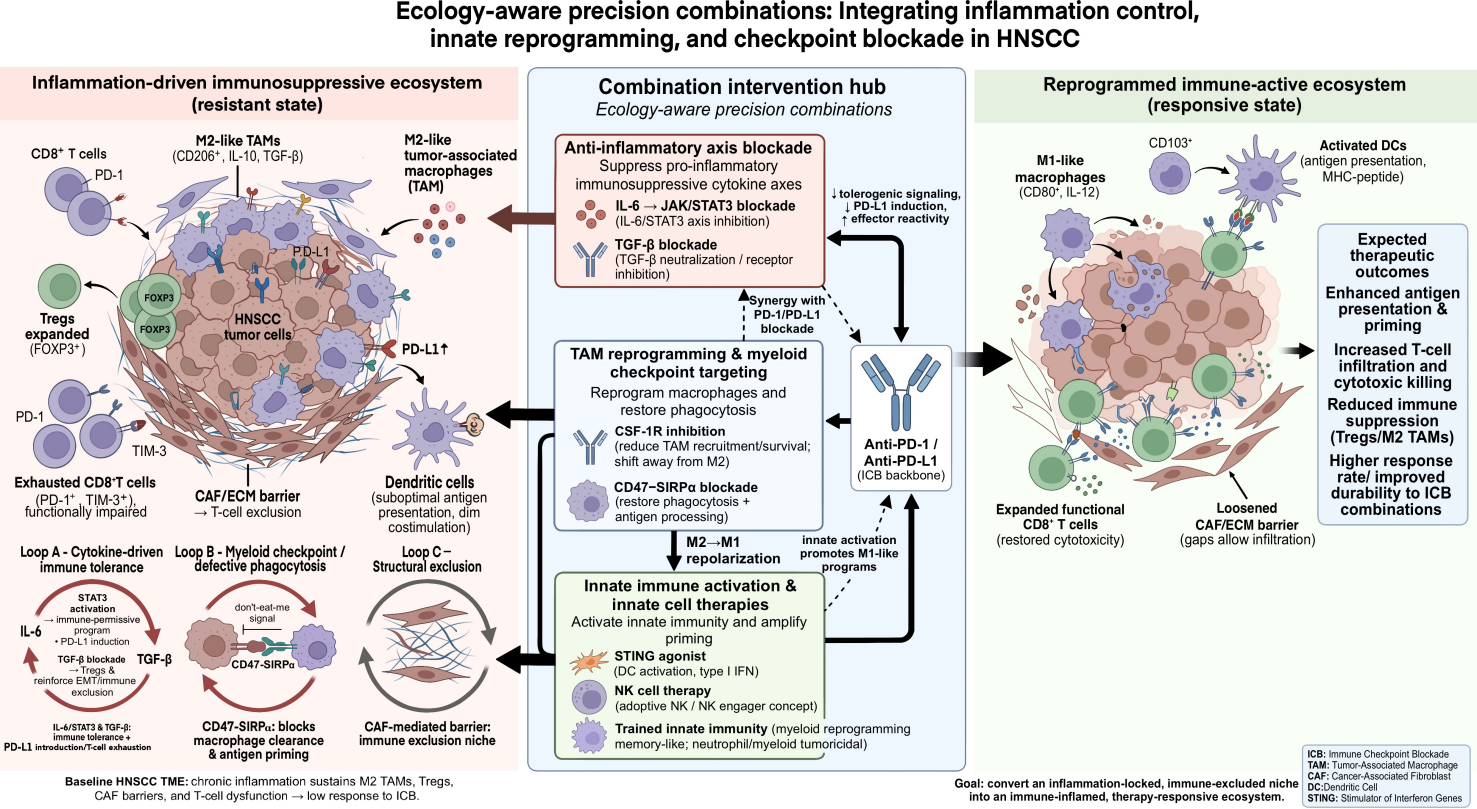
Ecology-aware precision combinations in HNSCC. The schematic illustrates the reprogramming of the TME from an inflammation-driven resistant state to an immune-active phenotype. (Left) Baseline resistance is sustained by IL-6/TGF-β mediated tolerance, CD47–SIRPα myeloid checkpoints, and CAF-mediated exclusion. (Center) PD-1/PD-L1 blockade serves as the therapeutic backbone, integrated with three synergistic modules: (1) anti-inflammatory blockade; (2) myeloid reprogramming (CSF-1R, CD47); and (3) innate immune activation (STING, NK). (Right) This multimodal strategy remodels the TME, promoting M1 macrophage polarization, enhanced antigen presentation, and restored cytotoxic T-cell infiltration.

Ultimately, all of these advances point toward a more “ecology-aware” treatment paradigm for HNSCC- one in which therapies are designed to read and reprogram the tumor’s inflammatory and immune landscape, converting the very drivers of immune escape into vulnerabilities that can be clinically exploited (**Figure 3**).

The PFDN2–CD64/FCGR1A finding also expands the therapeutic concept of myeloid reprogramming from local macrophage-targeted intervention to systemic protein–immune cell regulation. Although still exploratory, this axis suggests that circulating proteomic regulators may help identify or modify monocyte-driven inflammatory states before they become entrenched as immunotherapy-resistant niches (46).

## 7 Discussion

In HNSCC, chronic inflammation functions as an active, stage-dependent driver that connects malignant transformation to immunotherapy outcomes. Rather than acting through isolated pathways, inflammatory cues arising from carcinogen exposure, HPV-related disease, dysbiosis, and oxidative stress converge on interconnected NF-κB, STAT3, and TGF-β programs whose biological effects change over time. In early mucosal injury and premalignant transformation, NF-κB-centered signaling can amplify cytokine release and innate immune recruitment, while STAT3 activation supports epithelial survival and inflammatory tolerance. As lesions progress toward invasion, TGF-β increasingly interacts with NF-κB/STAT3-linked myeloid and stromal programs to promote EMT-like plasticity, CAF activation, extracellular matrix reprogramming, and immune exclusion. In treatment-exposed tumors, the same signaling network can become a resistance-maintaining circuit, sustaining checkpoint ligand expression, suppressive myeloid recruitment, impaired antigen presentation, and poor T-cell penetration. Consequently, inflammation does not simply coexist with cancer; it evolves from an initiating injury response into a suppressive tumor ecology that shapes whether ICB can generate effective and durable responses.

However, this framework should not be interpreted as implying a uniform or linear effect of inflammation across all HNSCC cases. Apparent inconsistencies among studies may arise from differences in anatomical subsite, HPV status, smoking and alcohol exposure, microbiome composition, prior treatment, and sampling time point. For example, inflammatory signatures may correlate with immune activation in some HPV-positive or T-cell-inflamed tumors, whereas similar cytokine or myeloid programs may reflect immune suppression, stromal exclusion, or tissue repair in HPV-negative, smoking-associated, or previously treated tumors. Thus, the biological meaning of an inflammatory marker depends strongly on cellular source, spatial localization, disease stage, and therapeutic context. This context-dependence explains why single biomarkers or pathway readouts often show inconsistent prognostic or predictive value across cohorts.

A key implication of this synthesis is that inflammatory pressure stabilizes suppressive ecosystems, often through myeloid skewing, fibroblast- and matrix-driven immune exclusion, metabolic constraint, and T-cell exhaustion. These features can create barriers to immune infiltration and function, explaining why many tumors exhibit an immune-excluded or immune-dysfunctional phenotype despite detectable immune activity. At the same time, inflammation can generate therapeutic vulnerabilities, suggesting that optimal strategies should rewire– rather than indiscriminately suppress– inflammatory circuits.

The failure of ICB in many patients is therefore unlikely to be explained by PD-1/PD-L1 expression alone. Resistance may occur when effector T cells fail to enter tumor nests, when antigen presentation is impaired, when suppressive myeloid cells dominate the local cytokine milieu, or when CAF- and extracellular matrix-driven barriers physically separate lymphocytes from malignant cells. In addition, chronic inflammatory signaling may simultaneously up-regulate checkpoint ligands and induce T-cell dysfunction, meaning that the presence of a checkpoint target does not necessarily indicate a reversible immune state. These observations support a more cautious interpretation of “inflamed” tumors: inflammation may represent productive anti-tumor immunity, compensatory immune suppression, or tissue-reprogramming programs that ultimately protect the tumor.

Single-cell and spatial profiling offers a practical bridge from mechanism to clinical translation by resolving composite “hot vs cold” labels into actionable micro-states and spatial neighborhoods. These tools can prioritize which inflammatory programs are upstream drivers of resistance, identify candidate biomarkers for patient stratification, and guide rational combinations that pair ICB with modules targeting myeloid suppression, stromal exclusion, or innate immune activation.

Several methodological and translational limitations should also be acknowledged. Preclinical models can reveal causal mechanisms, but they may not fully reproduce the etiologic diversity, microbial exposure, stromal architecture, and treatment history of human HNSCC. Retrospective human cohorts provide clinically relevant associations but are vulnerable to selection bias, heterogeneity in tissue sampling, and incomplete control of prior therapy, anatomical subsite, HPV status, and tobacco/alcohol exposure. Single-cell and spatial profiling have greatly refined our understanding of tumor ecology, but these approaches are often cross-sectional, sample limited regions of heterogeneous tumors, and may be affected by tissue dissociation bias, batch effects, platform-specific resolution, and inconsistent cell-state annotation. Therefore, many inflammation-related pathways identified by omics studies should be considered hypothesis-generating rather than definitively causal until validated by perturbation experiments, longitudinal sampling, and prospective clinical cohorts. Future studies should distinguish correlation from causation, define whether inflammatory programs are drivers or consequences of immune resistance, and test whether targeting these programs improves clinically meaningful outcomes beyond biomarker modulation.

Overall, an “ecology-aware” framework– integrating inflammatory drivers, TIME architecture, and high-resolution biomarker readouts– may enable more precise combination strategies that dismantle dominant inflammatory barriers while preserving effector immunity, ultimately improving the depth and durability of immunotherapy benefit in HNSCC.
